# Tracheostomy Practice Questionnaire: Development of a Valid and Reliable Tool for Assessing Tracheostomy Practice

**DOI:** 10.5339/qmj.2022.17

**Published:** 2022-03-16

**Authors:** Saja AlMarshad, Abdulaziz AlEnazi, Amani Owaidah

**Affiliations:** ^1^Department of Respiratory Care, Collage of Applied Science AlMaarefa University, Diriyah, 13713, Riyadh, Saudi Arabia E-mail: Smarshad@mcst.edu.sa; ^2^Department of Otolaryngology -Head and Neck surgery, Imam Abdul Rahman bin Faisal University, King Fahd Hospital of the University (KFHU), Khobar, KSA; ^3^Imam Abdulrahman bin Faisal University, Collage of Applied Medical Sciences, Clinical Laboratory Sciences Department, Dammam, Saudi Arabia

**Keywords:** Tracheostomy, questionnaire, practice, evidence-based, PS/QI

## Abstract

Background: Tracheostomy is among the oldest and most common surgical procedures for critically ill patients. Over the past decade, tracheostomy practice has changed regarding its indication, timing, technique, decannulation, and follow-up procedures. A systematic assessment tool for tracheostomy could maximize the quality of care and improve patient outcomes. This study develops a tool for systematically evaluating tracheostomy-related practices, assesses its validity and reliability, and conducts pilot testing of the tool.

Methods: The questionnaire development process involved three rounds using the Delphi technique with eight experts in airway management. The experts were selected from multiple healthcare specialties and workplace backgrounds. There was a two-week interval between each discussion round. In February 2019, the questionnaire themes and statements were identified through qualitative content analysis. Subsequently, in March 2019, the developed tool was emailed to 31 heads of tracheostomy care teams at multiple national hospitals for further validity and reliability assessment.

Results: The developed tool demonstrated reliability of 0.975. Tracheostomy-related practices showed acceptable levels in all 31 assessed hospitals with areas for improvement in the long-term follow-up domain.

Conclusion: This study designed a tool for the comprehensive assessment of tracheostomy-related practices. It can be used to monitor institutional outcomes, which can reduce costs. Moreover, this tool can be employed to track the improvement or deterioration of tracheostomy-related procedures and long-term follow-up to facilitate institutional progress. In addition, this tool could be used for formative and summative assessments of tracheotomy practices at national and international levels.

## Introduction

Tracheostomy has been practiced for over 3000 years and has been referred to as an incision into the “windpipe.”^1^ Until the twentieth century, tracheostomy was only considered in emergencies, including upper airway obstruction and asphyxia.^2^ However, it has since evolved and is performed on patients diagnosed with acute respiratory failure in the intensive care unit (ICU).^3^ Tracheostomy can be performed using a surgical or percutaneous approach, each with its indications and techniques.^4^ Tracheostomy procedure development, and the different available approaches and techniques, warrant attention toward outcome assessment to ensure safe practices.^5–7^ Currently, tracheostomy has been used to improve ventilation in many chronic long-term patients.^8–10^ Consequently, there is an increased need for care provision for in-home tracheostomized patients.^11^ This requires evaluating the tracheostomy practice during the acute stage and conducting an improved evaluation and follow-up of long-term patients using a standardized approach.^11–13^ Furthermore, there is a need to assess outcomes at all the stages of the procedure, including initiation, weaning, and long-term follow-up.

The use of tools focusing on patients’ outcome measures, such as quality of care, clinical effectiveness, and intervention efficacy, is increasing. These tools enable the identification and interventions in areas with reduced quality of care.^14–16^ A tracheostomy practice questionnaire could allow a quantitative and more comprehensive assessment of tracheostomy practices, facilitating improved monitoring of patient care. Currently, no instruments for evaluating tracheostomy practices exist. This study develops a tool for evaluating tracheostomy practices from initiation and weaning to long-term follow-up.

## Methods

### Study design

This study developed the Tracheostomy Practice Questionnaire (TPQ) in four steps: (1) the definition of the constructs/domains of tracheostomy practices; (2) the generation of behavioral items for representing each domain; (3) pilot testing of the TPQ; and (4) finalizing the scale based on the data collected in Step 3.

Steps 1 and 2 were performed through qualitative content analysis using the Delphi technique, a tool used to develop an expert-based judgment about a question^17^ to yield consensus within a selected expert panel.^18^ The study was ethically reviewed and approved by the Institutional Review Board of Almaarefa University.

### Steps 1 and 2 using the Delphi technique

### Participants and inclusion criteria

The study participants were experts in airway management and were selected from various health care specialties. The selection of study participants was not randomized; instead, it was purposive to represent relevant groups.^19^ The expert inclusion criteria included a minimum of five years of experience in airway management, being employed in a tertiary hospital, and being a member of a tracheostomy care team. This study selected eight experts: three ENT physicians, three respiratory therapists, and two nurses. All experts provided voluntary consent to participate in the current research and committed to the study from July to August 2018.

### Delphi rounds

Using the Delphi technique has many advantages and involves a select group about this important topic. Such advantages include providing a more comprehensive range of knowledge and experience. Debating may challenge thoughts and stimulate new ones, and having a group consensus may be more credible.^20^ It was reported in the evidence that by using the Delphi techniques, the experts could concentrate on rating, reviewing, and offering remarks on the items presented without the interference normally associated with more traditional face-to-face focus groups.^21^

The first round of the Delphi technique began with sending individual emails to all experts that contained open-ended questions regarding tracheostomy care practices. These questions, which were developed and validated by the authors of this study to address tracheostomy care practices, were:

[1] What is your opinion regarding the ideal process of tracheostomy practice within an institution?
[2] Describe your experience in managing tracheostomized patients?
[3] Mention your recommendation for informing tracheostomy practice in your institution from insertion to decannulation.
[4] What resources are required to achieve the recommendation for enhancing tracheostomy practice?

The experts were allowed two weeks to respond. Subsequently, the results were qualitatively analyzed by identifying similar statements among the experts. Affinity diagrams were used to group similar statements. Three main domains were extracted from the data collected at this stage, and other unique statements were grouped into allocated questionnaire themes. Finally, all similar and unique statements were categorized into themes and returned to the experts in the second round of Delphi.

In the second round, the experts were asked to rank the items generated from the first round of the Delphi technique from 1 to 10 based on priority. The major statistics used in Delphi studies are measures of central tendency and levels of dispersion (standard deviation and interquartile range) to present information concerning the collective judgments of respondents.^22^ Group consensus was statistically set at a mean value of ≥ 8 with an interquartile range (IQR) of 3 (9-6) based on previously reported methodology.^23^ Statements that did not reach consensus were grouped and returned to the experts in the third round to allow them to review and confirm responses. Moreover, in the third round, the entire group's responses were included to allow the experts to reconsider their responses based on those of others. All statements that reached consensus were included in the final tool draft within the three main domains.

Steps 3 and 4: Pilot testing and item generation of the tracheostomy practice questionnaire

The developed tool was named the TPQ. This tool comprised three main themes: tracheostomy procedure practice (14 statements), tracheostomy weaning protocol (9 statements), and tracheostomy long-term follow-up practice (7 statements). Each statement was rated on a 5-point Likert scale, with the possible responses being strongly agree (5), agree (4), unsure (3), disagree (2), or strongly disagree (1). To validate the TPQ, the questionnaire was sent to the heads of 31 tracheostomy care teams in national hospitals across Saudi Arabia as part of a pilot study. Participation in the pilot study was voluntary and anonymous.

The raw scores were coded, calculated, and analyzed using SPSS for Windows statistical package version 23. Descriptive statistics were reported, including the sum scores for each of the three domains and the total score for the TPQ.

## Results

### Descriptive statistics

The pilot sample included the heads of 31 tracheostomy care teams; 13 were from government hospitals, 10 from semi-government hospitals, five from university hospitals, and three from private hospitals. Most heads of tracheostomy care teams were ENT specialists (n = 9, 29%). Most study participants were aged 30–40 years.

### Validity and reliability of TPQ

A survey instrument should be both reliable and valid. We assessed face validity by emailing the developed tool back to the experts to obtain their input regarding the questionnaire's validity.

Reliability was assessed based on internal consistency after obtaining data from the pilot sample. Cronbach's alpha, which is widely used in social science research to estimate the internal consistency of the reliability of a measurement within a scale^24^ and which can range from 0.0 to 1.0, was used. It quantifies the degree to which items on an instrument are correlated with one another.^25^ Thus, its use assured that the items within the subscales on the TPQ each measured their respective construct and confirmed their correlation with the other items within the subscale.^26^ Reliability was considered acceptable when Cronbach's alpha exceeded 0.8. The questionnaire showed reliability with a Cronbach's alpha of 0.97. Moreover, we calculated item-total correlations that measured internal consistency and compared the individual item scores with the total scale score. All included statements had item-total correlations >0.4, and the others were considered for rejection.

### TPQ pilot study results

The questionnaire results were interpreted based on two major factors: the sample size, the sum value of the overall scores, and the sum score for each TPQ domain.

### The first domain

This domain, the tracheostomy procedure practice domain, included 14 statements (Appendix 1). The sum score for each domain was indicative of the tracheostomy procedure quality. The maximum score for the tracheostomy care practice domain for the pilot sample was 2170. This could be produced with a score of 5 for all 14 statements by each of the 31 study participants. The maximum score reflected an ideal practice. We obtained a total score of 1664 (76.68%) (>50% and < 80%), indicating an acceptable practice. Table 1 summarizes the results of the tested samples.

### The second domain

The second domain, the tracheostomy weaning protocol, included nine statements (Appendix 1). This domain focused on gradually weaning patients off tracheostomy and had a maximum possible score of 1395. This corresponds to a score of 5 for all statements by each sampled hospital, reflecting an ideal practice in this domain (80%–100%). The maximum score in our sample was 970, reflecting a percentage of 69.53% (>50% and < 80%) and indicating an acceptable practice in this domain.

### The third domain

The third domain, tracheostomy long-term follow-up practice, included seven statements (Appendix 2). This domain focused on assessing the long-term follow-up process for tracheostomized patients after discharge. Moreover, home care services provided were assessed within this domain. The maximum score of this domain was 1085 across the 31 hospitals, corresponding to a score of 5 for all statements by each sampled hospital and reflecting an ideal practice. In our sample, the total score for this domain was 742, reflecting a percentage of 68.38% (>50% and < 80%) and indicating an acceptable practice. Table 2 presents the interpretation of our sample results.

### Overall assessment of the TPQ

The total score was obtained as the sum of the scores of the 30 questionnaire items within the tested sample. The possible score was grouped into three categories. A sum score < 50% indicated that tracheostomy required major improvement areas in the first category. Furthermore, a score of >50% and < 80% was indicative of acceptable tracheostomy care practices with areas of improvement (statements had a mean score < 2). Finally, abetween 80% and 100% indicated an acceptable tracheostomy practice.

In our sample, the sum score was 3376 (72.4 %), which indicated acceptable tracheostomy practices with areas of improvement, summarized in Table 2.

## Discussion

Tracheostomy is a common surgical procedure that is crucial in airway management.^18,19,27,28^ Monitoring tracheostomy care practice is essential for improving outcomes in tracheotomized patients.^29^ Due to the enhancement in technologies and medical care, more patients with chronic illnesses are being sent home with long-term tracheostomies.^30^ Long-term tracheostomy is not well defined in the literature. This study refers to any patient sent home with a tracheostomy as a long-term tracheostomized patient. This study describes the development and validation of a tool for assessing clinical practice from initiation till weaning of the tracheostomized patients. It was generated so that it can be applied to any age category using the Delphi technique. A thorough evaluation of the available literature and acknowledgment of the issues of the Delphi technique, such as self-interest and the potential biases this might lead to,^31^ were performed. However, the authors chose this technique to generate the items of the standardized tool to overcome the variability of the published evidence regarding the evaluation of tracheostomy-related practices since many international surveys reported institutional practices without evidence-based benchmarking to current ideal practices.^32,33^ The Identification of standardized practices proven to improve patients’ outcomes and adherence to hospital follow-ups could be of great clinical utility.^34,35^

The TPQ is a 30-item questionnaire for quantitatively describing tracheostomy-related practices. It comprises three main domains: the tracheostomy procedure, tracheostomy weaning protocol, and tracheostomy long-term follow-up, which were identified as being the most valuable based on our qualitative data analysis.

The TPQ can monitor institutional outcomes, which can be applied to reduce costs. Moreover, determining the weaknesses and strengths of institutional practices can optimize strategic plans for improving tracheostomy care outcomes at the national level. The domains of the tracheostomy procedure, weaning protocol, and long-term tracheostomy follow-up practice should be regularly evaluated to improve the quality of care provided to patients undergoing tracheostomy. Future studies, including all hospitals at the national and international levels, need to establish a baseline for comparison and future improvement plans.

This study was limited by our inability to recruit experts from many disciplines. We acknowledge the value of other disciplines as experts within tracheostomy care teams, such as speech and language pathologists. However, because of the voluntary participation, we could not recruit experts within that specialty. The TPQ must be tested on a larger scale to reassess its reliability within different settings.

## Conclusion

The TPQ developed is a reliable tool that can identify areas of reduced quality of care in tracheostomy care practices. This tool can be utilized to evaluate the adherence extent of medical centers to optimal practice and regular follow-up of the improvement progress.

## Figures and Tables

**Table 1:** Pilot study TPQ scores

Domain	Pilot study			Calculated ranges	
	Score	Percentage	Range	Percentage	Interpretation
The tracheostomy procedure practice (14 statements)	1664	76.68%	1736–2170	80%–100% > 50%– < 80% < 50%	Ideal Acceptable with areas of improvement Major improvement required
The tracheostomy weaning protocol (9 statements)	970	69.53%	1116–1395	80%–100% >50%– >80% < 50%	Ideal Acceptable with areas of improvement Major improvement required
Tracheostomy long term follow-up practice (7 statements)	742	68.38%	868–1085	80%–100% >50%– <80% < 50%	Ideal Acceptable with areas of improvementMajor improvementrequired
Total score (the sum of all domains)	3376	72.60%	3720–4650	80%–100% >50%– <80% < 50%	Ideal Acceptable with areasof improvement Major improvement required

**Table 2:** Areas of improvement in the hospitals evaluated.

Statement	Mean value

Statement 14: We consider different cuff designs for minimizing mucosal trauma.	1.8

Statement 19: Speech pathologists have a clear role in the tracheostomy care team.	1.9

Statement 27: We provide regular home visits for tracheostomy patients.	1

Statement 30: The tracheostomy weaning is conducted after discharge.	0.9


**Table Appendix 1:** Copy of the tracheostomy practice questionnaire (TPQ)

Questionnaire statement	Not at all 1	To a small extent 2	To some extent 3	To a moderate extent 4	To a large extent 5

Domain 1: Tracheostomy procedure					

1. Tracheostomy procedure is well regulated in our practice					

2. Only the authorized physicians per protocol are allowed to perform tracheostomy					

3. There is a clear policy regarding the tracheostomy procedure					

4. It is common to perform percutaneous tracheostomy in our ICUs					

5. A routine tracheostomy change is always done by a competent staff					

6. Emergency equipment, including BMV with a mask, obturator, dilator, and extra tracheostomy tube, are always available at the bedside of the tracheotomized patient					

7. My role is very clear to me in the care of tracheotomized patients					

8. The first tracheostomy change is performed only by the operating physician or equivalent					

9. The most frequent timing for performing tracheostomy procedure post-tracheal intubation is between 15-21 days					

10. The first tracheostomy change was performed after one-two weeks					


## References

[18] http://dx.doi.org/10.7275/rrph-t210.

[20] http://dx.doi.org/10.1080/0142159x.2017.1245856.

[21] http://dx.doi.org/10.5959/eimj.v9i3.548..

[22] http://dx.doi.org/10.1046/j.1365-2648.2000.t01-1-01567.x.

[26] http://dx.doi.org/10.1002/nur.4770130612.

[27] http://dx.doi.org/10.1097/01.ccm.0000145232.46143.40..

[28] http://dx.doi.org/10.1097/01.ccm.0000150026.85210.13..

[31] http://dx.doi.org/10.1016/j.techfore.2011.07.008.

